# Clinical characteristics and outcomes of infants compared with children diagnosed with rhabdomyosarcoma: Analysis of surveillance, epidemiology and end results data from 2000 to 2016

**DOI:** 10.1002/cnr2.1503

**Published:** 2021-07-13

**Authors:** Hannah D. Rees, Nancy K. Hills, Amit J. Sabnis, Asmin B. Tulpule, Tom K. Shimotake, Robert E. Goldsby

**Affiliations:** ^1^ Pediatric Hematology/Oncology UCSF Benioff Children's Hospital San Francisco California USA; ^2^ Department of Epidemiology and Biostatistics University of California San Francisco California USA; ^3^ Pediatric Neonatology UCSF Benioff Children's Hospital San Francisco California USA

**Keywords:** infants, pediatric cancer, sarcoma

## Abstract

**Background:**

Rhabdomyosarcoma (RMS) is the most common soft‐tissue sarcoma of childhood, but occurs infrequently in infants (<1 year). Historically, infants with RMS have worse overall survival compared to other pediatric age groups.

**Aim:**

This study aims to assess the clinical features and treatment factors associated with survival comparing infants to children aged 1–9 years diagnosed with RMS.

**Methods:**

Children aged <10 years diagnosed with RMS between 2000 and 2016 were identified using the SEER database. Descriptive statistics were used to assess demographic, clinical, and treatment characteristics of infants and children with RMS. Kaplan–Meier estimates and Cox proportional hazards regression were performed to assess for factors associated with survival.

**Results:**

Age <1 year was independently associated with an increased risk of mortality. Compared to children aged 1–9 years, fewer infants received standard of care therapy, that is, chemotherapy combined with local control (surgery and/or radiation; 86.8 vs. 75.7%; *p* = .009). In comparing the frequency of specific treatment modalities (used alone or in combination with other modalities), infants were less likely to receive radiation therapy (34.0 vs. 66.4%; *p* < .001) and more likely to receive surgery (68.9 vs. 57.5%; *p* = .02) than children aged 1–9 years. Across age groups, chemotherapy combined with local control was significantly associated with reduced mortality. Alveolar histology, metastatic disease, and Hispanic ethnicity were negatively associated with survival.

**Conclusions:**

Age of <1 year was an independent risk factor for increased mortality from RMS compared to ages 1–9 years. Fewer infants were treated with chemotherapy combined with local control, the therapy associated with best survival in all age groups. Other factors contributing to differences in survival should be further explored.

## 1 INTRODUCTION

Rhabdomyosarcoma (RMS) is a malignancy characterized by myogenic differentiation and is the most common soft‐tissue sarcoma among children and adolescents.^1^ RMS primarily affects children under 10 years of age (~70% of cases) and the incidence decreases with increasing age.^2^ Age, extent of disease, primary site, tumor histology and FOXO1 fusion status all impact survival.^3^, ^4^, ^5^, ^6^ Regardless of prognostic features, curative treatment for RMS includes systemic chemotherapy and local control using surgery, radiation therapy (XRT), or both.^2^, ^7^

Prior studies show age at diagnosis of RMS influences risk for treatment failure, with children <1 year and >10 years having worse outcomes than those aged 1–9 years.^5^, ^6^, ^8^, ^9^ In general, children aged 0–12 years have better prognoses than those 13–19 years when assessing all histological subtypes of RMS.^5^ Compared to younger patients, those over the age of 13 with RMS have distinct biological and clinical features and, on average, have been found to present with larger primary tumors of different distribution of sites, higher frequency of metastatic disease at diagnosis, greater prevalence of alveolar histology, higher incidence of fusion positivity, and disease that may be less responsive to standard therapies.^3^, ^4^, ^10^, ^11^, ^12^, ^13^, ^14^, ^15^

There is limited data explaining why infants with RMS fare poorly compared to older children. Some studies suggest that disparities in outcomes between infants <1 year and older children may be in part due to the absence of local disease control.^16^, ^17^ We utilized population‐level data from the surveillance, epidemiology, and end results (SEER) program to assess clinical features and treatment factors associated with survival, focusing on infants and comparing them to children aged 1–9 years diagnosed with RMS in the modern era.

## 2 MATERIALS AND METHODS

### 2.1 Patients

The data for this retrospective cohort study were obtained from the National Cancer Institute's SEER Program.^18^ The SEER program's data on cancer incidence and survival from population‐based cancer registries covers 35% of the U.S. population. It is an U.S.‐based database that provides initial stage at diagnosis and survival data for patients with cancer. Other data collected by the SEER program include patient demographics, primary tumor site, tumor morphology and first course of treatment.^8^, ^18^

Children aged <10 years diagnosed with RMS between 2000 and 2016 in the SEER 18 Regs Custom Dataset were included. Patients were dichotomized into age groups including infants (aged <1 year) and children aged 1–9 years. The age cutoff of <1 year was selected given literature indicating that infants with RMS experience worse outcomes than older children.^5^, ^8^, ^9^, ^16^, ^17^, ^19^ Primary tumor sites were grouped in accordance with anatomic site designation utilized by the Children's Oncology Group.^20^ For staging, a variable was created to consolidate multiple SEER outputs including *Summary Stage 2000, Combined Summary Stage 2000*, and *Historic Stage A*. Rarely, if there was a discrepancy in stage across the three variables, we included the stage consistent across two variables. If a given entry included a discrepancy between two variables regarding local versus regional or distant versus regional, local, or distant was selected given that those stages would most affect therapy.

Patient demographic and clinical characteristics were compared between children aged <1 year and those 1–9 years. These variables included sex; race and ethnicity (Hispanic, Black, white, and other [American Indian/Alaska Native, Asian/Pacific Islander, Native America, and unknown]); primary tumor site (orbit, parameningeal, head and neck, trunk including liver and kidney, genitourinary, extremities, and unknown); histology (alveolar, embryonal/other [pleomorphic, spindle, mixed, ganglionic differentiation, and not otherwise specified]); and stage (localized, regional, distant, and unknown). Tumor size was not included in the analysis due to high rates of missing data.

Treatment data indicated whether each patient received surgery (surgery, no surgery, and unknown); chemotherapy (chemotherapy, no/unknown); and radiation (radiation, no/unknown). The grouping of treatment status (no and unknown treatment) was determined by provided SEER data. Because 86% of children received more than one modality of treatment, we categorized treatment data as (a) none/local control only (b) chemotherapy only or (c) chemotherapy combined with local control (surgery, radiation, or both). These groupings were made to compare rates of standard treatment for RMS (including chemotherapy with local control measures) to partial treatment (chemotherapy alone or local control alone or no therapy) between the two age groups, and assess for associations between treatment type and mortality.

Overall survival time in months was calculated with SEER diagnosis date as time origin, with surviving children censored at the date of last follow‐up. Children who died within a month of diagnosis were assigned a survival time of 0.5 months.

### 2.2 Statistical analysis

Group differences regarding patient and tumor characteristics were compared using chi‐square tests or Fisher's exact tests where appropriate. Kaplan–Meier curves were used to compare the shape of the survival functions for infants and children aged 1–9; these were compared using the log‐rank test. We examined the association of mortality with patient characteristics, including age group, in univariate Cox proportional hazards models. We then constructed models in which age group was adjusted for each of the other characteristics found to be significantly associated with mortality at the 0.05 level in univariate analysis. Finally, a multivariable regression model was constructed including covariates that remained independently associated with mortality when included in a model with age group. The SEER database was accessed using SEER*Stat, version 8.3.8. All statistical analyses were performed using STATA, version 16.1 (College Station, TX).

## 3 RESULTS

A total of 1154 children with RMS diagnosed before 10 years of age were reported to SEER between 2000 and 2016, and of these, 103 were <1 year of age at diagnosis. The clinical characteristics of this study population according to age <1 year and between 1 and 9 years at time of diagnosis are shown in Table 1. There were no significant differences between the age groups in terms of sex, race/ethnicity, year of diagnosis, primary tumor site, histology, or extent of disease.

**TABLE 1:** Demographic and clinical characteristics

Characteristic	All patients (*N* = 1154)		Age <1 year (*n* = 103)		Age 1–9 years(*n* = 1051)		*p* Value*
	*n*	(%)	*n*	(%)	*n*	(%)	
Sex							.69
Female	492	(42.6)	42	(40.8)	450	(42.8)	
Male	662	(57.4)	61	(59.2)	601	(57.2)	
Race/Ethnicity							.35
Black	168	(14.6)	13	(12.6)	155	(14.7)	
Hispanic	331	(28.7)	30	(29.1)	301	(28.6)	
White	561	(48.6)	47	(45.6)	514	(48.9)	
Other	94	(8.1)	13	(12.6)	81	(7.7)	
Primary tumor site							.43**
Orbit	97	(8.4)	9	(8.7)	88	(8.4)	
Parameningeal	106	(9.2)	4	(3.9)	102	(9.7)	
Head/Neck	247	(21.4)	24	(23.3)	223	(21.2)	
Trunk	347	(30.1)	33	(32.0)	314	(29.9)	
Genitourinary	205	(17.8)	20	(19.4)	185	(17.6)	
Extremities	132	(11.4)	10	(9.7)	122	(11.6)	
Other/Unknown	20	(1.7)	3	(2.9)	17	(1.6)	
Histology							.86
Alveolar	261	(22.6)	24	(23.3)	237	(77.5)	
Embryonal/Other	893	(77.4)	79	(76.7)	814	(22.5)	
Stage							.22
Localized	428	(37.1)	41	(39.8)	387	(36.8)	
Regional	390	(33.8)	37	(35.9)	353	(33.6)	
Distant	289	(25.0)	18	(17.5)	271	(25.8)	
Unknown	47	(4.1)	7	(6.8)	40	(3.8)	

^*^ *p*‐Values calculated using chi‐square test unless otherwise indicated.

^**^ *p*‐Value calculated using Fisher's exact test.

In comparing the frequency of specific treatment modalities (used alone or in combination with other modalities), infants were more likely to receive surgery (68.9 vs. 57.5%; *p* = .02), and less likely to receive radiation (34.0 vs. 66.4%; *p* < .001) and chemotherapy (92.2 vs. 95.9%; *p* = .08), compared to children diagnosed with RMS between 1 and 9 years of age. Standard therapy for rhabdomyosarcoma includes both chemotherapy and local control; however, infants were less likely to receive standard of care treatment (chemotherapy and local control: 75.7 vs. 86.8%) and more often treated with chemotherapy alone (16.5 vs. 9.1%) or with either local control alone or no therapy (7.8 vs. 4.1%; *p* = .009, Table 2).

**TABLE 2:** Treatment differences by age

Treatment	All patients (*N* = 1154)		Age <1 year (*n* = 103)		Age 1–9 years(*n* = 1051)		*p* Value*
	*N*	(%)	*n*	(%)	*n*	(%)	
Individual treatment modalities							
Surgery							.02
No surgery	471	(40.8)	31	(30.1)	440	(41.9)	
Any surgery	675	(58.5)	71	(68.9)	604	(57.5)	
Unknown	8	(0.7)	1	(1.0)	7	(0.7)	
Chemotherapy							.08
No/unknown chemo	51	(4.4)	8	(7.8)	43	(4.1)	
Any chemotherapy	1103	(95.6)	95	(92.2)	1008	(95.9)	
Radiation							<.001
No/unknown radiation	421	(36.5)	68	(66.0)	353	(33.6)	
Any radiation	733	(63.5)	35	(34.0)	698	(66.4)	
Combined treatment							
Incomplete treatment	51	(4.4)	8	(7.8)	43	(4.1)	.009**
No treatment	28	(2.4)	7	(6.8)	21	(2.0)	
Surgery only	20	(1.7)	1	(1.0)	19	(1.8)	
XRT only	1	(0.1)	0		1	(0.1)	
XRT + surgery	2	(0.2)	0		2	(0.2)	
Chemo only	113	(9.8)	17	(16.5)	96	(9.1)	
Chemo + local control	990	(85.8)	78	(75.7)	912	(86.8)	
Chemo + radiation	337	(29.2)	8	(7.8)	329	(31.3)	
Chemo + surgery	260	(22.5)	43	(41.7)	217	(20.6)	
Chemo + radiation + surgery	393	(34.1)	27	(26.2)	366	(34.8)	

^*^ *p*‐Values calculated using chi‐square tests to compare individual treatment modalities by age group.

^**^ *p*‐Value calculated using chi‐square test comparing none/local control only versus chemo only versus chemo + local control by age group.

In univariate analysis, infants had 50% greater mortality compared to children ages 1–9. We identified additional factors beyond age that were associated with worse outcomes. Namely, we found that Hispanic children had a 38% higher rate of mortality than white children. Trunk, parameningeal, head/neck and extremity tumors were associated with worse outcomes when compared to genitourinary tumors, and children with alveolar histology were twice as likely to die as children with embryonal/other tumors. Children with tumors that were regional or distant were also significantly more likely to die than those with localized tumors (Table 3).

**TABLE 3:** Associations with time to mortality: Cox proportional hazards models

Characteristic	Univariate models			Final multivariable model		
	HR	95% CI	*p* Value	HR	95% CI	*p* Value
Age category						
1–9 years	Ref			Ref		
<1 year	1.52	(1.07, 2.15)	.02	1.69	(1.18, 2.42)	.005
Sex						
Female	Ref					
Male	0.97	(0.77, 1.21)	.78			
Race						
White	Ref			Ref		
Black	1.20	(0.86, 1.68)	.27	1.19	(0.84, 1.68)	.34
Hispanic	1.38	(1.06, 1.79)	.015	1.58	(1.05, 2.37)	.03
Other	1.54	(1.03, 2.30)	.035	1.32	(1.01, 1.72)	.04
Year of diagnosis						
2000–2008	Ref					
2009–2016	0.92	(0.73, 1.18)	.53			
Primary tumor site						
Orbit	Ref					
Genitourinary	1.31	(0.64, 2.71)	.45	1.13	(0.51, 2.52)	.76
Trunk	3.68	(1.92, 7.03)	<.001	2.51	(1.21, 5.23)	.01
Parameningeal	3.04	(1.49, 6.18)	.002	1.91	(0.98, 4.80)	.06
Head/Neck	3.31	(1.71, 6.39)	<.001	2.16	(1.02,4.57)	.04
Extremities	3.74	(1.89, 7.39)	<.001	1.87	(0.85, 4.09)	.12
Other/Unknown	3.75	(1.36, 10.31)	.01	2.91	(0.94, 9.03)	.06
Histology						
Embryonal/Other	Ref			Ref		
Alveolar	2.02	(1.60, 2.55)	<.001	1.61	(1.24, 2.09)	<.001
Stage						
Localized	Ref			Ref		
Regional	1.60	(1.16, 2.20)	.003	1.41	(1.02, 1.96)	.04
Distant	4.15	(3.08, 5.57)	<.001	3.20	(2.34, 4.36)	<.001
Treatment						
No treatment/local only	Ref					
Chemotherapy only	1.64	(0.96, 2.81)	.07	0.90	(0.46, 1.75)	.76
Chemotherapy + local	0.56	(0.34, 0.91)	.01	0.43	(0.23, 0.81)	.008

**FIGURE 1:**
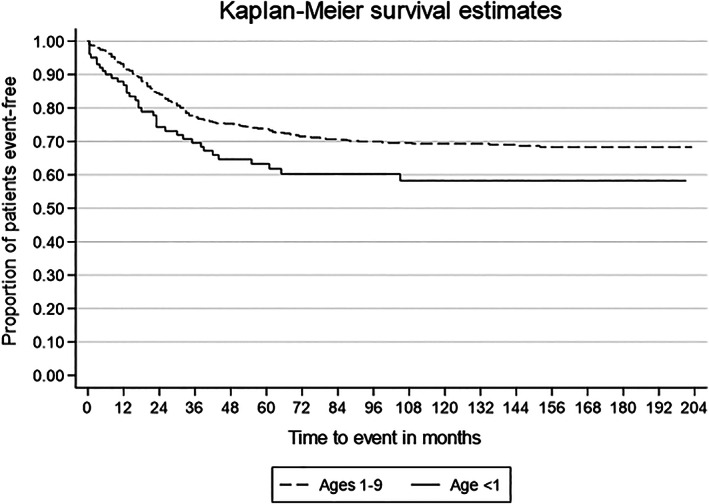
Kaplan–Meier survival comparing survival of patients diagnosed <1 year of age compared to patients diagnosed between 1 and 9 years of age

In models in which the association between age group and outcome was adjusted by each of the other covariates individually, age <1 year remained independently associated with higher rates of mortality when adjusted for race, tumor site, stage or histology. No evidence of interactions between age and any other factor was found. In a multivariable regression model, age <1 year, Hispanic ethnicity, tumor of the trunk and head/neck (compared to tumor of the orbit), alveolar histology, and metastatic disease, were significantly and independently associated with worse survival; treatment with chemotherapy and local tumor control was protective (Table 3). Five‐year overall survival was 63.3% for infants and compared to 73.7% for children diagnosed from 1 to 9 years of age (Figure 1) and the difference persists when adjusting for other prognostic and treatment factors.

## 4 DISCUSSION

In this study, we found that age less than 1‐year old at diagnosis of RMS conferred an additional risk of mortality that was independent of other factors, like histology, stage of disease and treatment. While RMS is rare in infants, standard therapies pose particular challenges, including the potential for higher risk of both acute and late effects of therapy. It is possible the rarity of cancer in infants, along with provider concerns regarding treatment toxicity, could impact timing of diagnosis, treatment decisions and, ultimately, outcome.

Prior studies indicate infants fare worse than older children diagnosed with RMS.^6^, ^8^, ^9^, ^16^, ^17^, ^19^ The overall estimated 5‐year failure free survival for infants with RMS varies from 42 to 57%. Infants with localized RMS treated on recent therapy trials have a 5‐year overall survival of 76% compared to 87% for children diagnosed with RMS between 1 and 9 years of age.^16^ In another study including children with metastatic and non‐metastatic disease enrolled in therapeutic trials in Italy between 1979 and 2001, the overall survival in infants was 61% compared to 67% for older children (including adolescents). Our report focuses on infants diagnosed with RMS from 2000 through 2016, whom we found to have five‐years survival rates of 63.3% compared to 73.3% for children aged 1–9 years at diagnosis.

Some of the variation in outcome relates to the differences in therapy approach. The most recent assessment of infants with RMS treated on IRS IV and V found that 30% had local failures.^16^ Additionally, of 72 infants treated on these protocols, 30 had major deviations from protocol‐specified radiation dose or volume. Similarly, the Children's Oncology Group determined that local failure among infants with RMS was associated with having received individualized, less than protocol‐recommended radiation therapy.^21^ While infants may be more susceptible to the toxic effects of standard therapy, our study, along with others, indicates the importance of combining local control with systemic chemotherapy to offer the best chance of cure.^22^, ^23^

While age <1 year was associated with significant variations in treatment administered, multivariate models found that age <1 year at diagnosis was associated with an increased risk for mortality independent of therapy received. This finding builds upon data from the Children's Oncology Group in 2011 which suggest that high rates of local failure among infants is likely attributable to infants receiving less than protocol‐specified local control.^16^ We also found infants are more often treated with sub‐standard therapy (chemotherapy alone, local control alone or no therapy). Although deviations from treatment protocol may be a contributing factor to worse outcomes among infants, our findings suggest that age <1 year is a prognostic factor independent of treatment received.

This finding may be influenced by a number of factors. First, the timing and doses of therapies can impact outcome.^24^, ^25^ It may be that the doses and/or types of chemotherapy were suboptimal or the doses and/or fields of radiation were inadequate. Additionally, there are no data regarding the extent of surgery or specifics of radiation for local control. Thus, treatment may have been sub‐optimal even in patients who were judged to receive standard of care therapy in our analysis. Second, it is possible the rarity of disease among infants may result in delayed diagnosis; however, the limited data available do not support this notion. The percentage of localized, regional, and metastatic disease, respectively, was similar for infants (39.8, 35.9, and 17.5%) and children (36.8, 33.6, and 25.8%). Third, there may be molecular differences between RMS in infants and older children.^26^, ^27^ Additionally, children with a history of RMS have a fivefold increased risk of developing second malignant neoplasms (SMN) compared to malignancy among the general population, suggesting that there is a higher rate of cancer predisposition gene alterations among this population.^28^, ^29^, ^30^, ^31^

Avoiding late effects in this vulnerable population is warranted. Studies of late effects in patients diagnosed with RMS demonstrate the risks of curative therapy.^28^, ^32^, ^33^, ^34^, ^35^ Survivors of RMS can develop hematologic or solid SMNs as a result of treatment‐related toxicity and those with cancer predispositions will be at the highest risk.^28^ Among children who receive radiation for head and neck RMS, the most common long‐term effects included facial growth retardation, neuroendocrine dysfunction, and visual problems.^34^, ^36^ Abdominal and pelvic radiation among children with paratesticular RMS has been associated with chronic diarrhea, urethral strictures, urethritis, and skeletal hypoplasia, whereas chemotherapeutics were linked to cystitis and gonadal dysfunction.^32^ Other studies have shown that infants are at higher therapy‐related risk than older children. Infants with Wilms' tumor have higher incidence of severe toxicity and therapy‐related mortality, and among infants with RMS, there is increased risk of chemotherapy‐associated hepatopathy.^22^, ^23^ Infants in our study were treated significantly less often with radiation. Radiation is known to be an important therapeutic tool in the arsenal against RMS. Withholding standard of care radiation in infants out of concern for late effects may have consequences on survival in this age group and must be considered with caution.

In addition to age, Hispanic ethnicity was found to be independently associated with worse survival. Previous population‐level studies have not found associations between race/ethnicity and outcomes among children with RMS. Baker et al. illustrated that although non‐white patients with RMS were more likely to present with invasive T2 tumors and positive regional lymph nodes compared to white patients, they had similar outcomes after adjusting for T stage, risk, and age.^37^ Survival disparities across race/ethnicity have been well documented for many other pediatric malignancies.^38^, ^39^ These disparities are thought to relate to differences in socioeconomic status, insurance coverage, time to diagnosis, enrollment on cooperative group trials, pharmacogenetic factors, and disease biology.^38^, ^40^ Future work should study the impact of social, structural, and biological factors on the outcomes of children with RMS to achieve equitable care across race/ethnicity.

Baker et al^19^ reported that non‐white patients with RMS were more likely to present with invasive T2 tumors (P ¼ 0.03), tumors with positive regional lymph nodes (N1, P ¼ 0.002), large tumors (>5 cm, P ¼ 0.006) and tumors which were stage 2 or 3 (P ¼ 0.03) compared with whites.

While the power of the SEER resource lies in its large numbers and rigor of the data, the SEER database has significant limitations. Namely, it does not include details regarding chemotherapy regimens, radiation dosing or surgical approaches. The focus of the SEER data is on first therapy and there is no information regarding relapse or salvage therapies. SEER data is limited in its reporting of tumor size and does not include traditional RMS Group and Stage information. SEER does not include any information regarding molecular profiling of cancers. As with all studies, caution is needed when analyzing race and ethnicity variables. In this analysis, the SEER variable used combines race and ethnicity and we are not able to separate for purposes of analysis. Additionally, factors that shape the course of treatment including patient family preferences, physician recommendations, comorbidities, and proximity to treatment providers, are not measured. The inclusion of these data in future analyses will be essential to furthering our understanding of survival differences among children with RMS.

In conclusion, this study shows that patients diagnosed with RMS <1 year of age have a worse prognosis compared to children ages 1–9 year, independent of treatment‐received. Children of all ages have significantly improved outcomes if they receive treatment that combines local control with chemotherapy. Given that infants are less likely to receive standard of care therapy than older children, this is likely a contributing factor to worse outcomes among this age group; however, other factors also contribute to the worse survival. Future investigations should assess whether there are molecular differences and/or other specific treatment differences that impact survival. We should also investigate the late effects in infants treated with standard therapy to garner a better understanding of the potential risks of late effects in this population. Perhaps newer, more targeted therapies will be less toxic for infants and allow for improved outcomes in this population.

## CONFLICT OF INTEREST

The authors declare there is no conflict of interest.

## AUTHOR CONTRIBUTIONS

All authors provided significant contributions. *Conceptualization, Data Curation, Formal Analysis, Investigation, Methodology, Project Administration, Resources, Software, Validation, Writing*—*Original Draft, and Writing*—*Review and Editing*, H.R.; *Conceptualization, Data Curation, Formal Analysis, Investigation, Methodology, Project Administration, Resources, Software, Supervision, Validation, Writing—Original Draft, and Writing—Review and Editing*, N.H.; *Formal Analysis, Methodology, Supervision, Writing—Original Draft, and Writing—Review and Editing*, A.S.; *Formal Analysis, Methodology, Supervision, Writing—Original Draft, and Writing—Review and Editing*, A.T.; *Writing—Original Draft and Writing—Review And Editing*, T.S.; *Conceptualization, Data Curation, Formal Analysis, Funding Acquisition, Investigation, Methodology, Project Administration, Resources, Software, Supervision, Validation, Visualization, Writing—Original Draft, and Writing—Review and Editing*, R.G.

## ETHICAL STATEMENT

Given that this study used de‐identified data from the SEER database and did not involve patient contact, this study was exempt from Institutional Review Board review.

## Data Availability

The data used in this study are freely accessible and can be obtained via the National Cancer Institute SEER database.
